# 

**Published:** 1955-09

**Authors:** 


### Contents

| | | Page |
|---|---|---|
| EDITORIAL | | 141 |
| COUSIN MARRIAGE | *Dr. J. A. Fraser Roberts* | 142 |
| MENINGITIS IN CHILDHOOD | *Dr. Beryl D. Corner* | 150 |
| MENINGITIS | *Dr. A. M. G. Campbell* | 162 |
| WHAT THE G.P. WANTS FROM A CASUALTY DEPARTMENT | *A General Practitioner* | 167 |
| CASUALTY DEPARTMENTS | *Mr. Herbert K. Bourns* | 168 |
| CASE REPORT—JAUNDICE WITH HEPATIC FAILURE | | 173 |
| NEWS FROM SOCIETIES<br>TORQUAY AND DISTRICT MEDICAL SOCIETY | | 176 |
| NOTES AND NEWS<br>UNIVERSITY OF BRISTOL | | 179 |
| APPOINTMENTS | | 180 |

